# Short-Term Trace Element Distribution Following Application of *Sargassum*-Based Liquid Biofertilizer in a Soil–Plant–Tomato Fruit System

**DOI:** 10.3390/plants15060901

**Published:** 2026-03-14

**Authors:** Yaset Rodríguez-Rodríguez, Máximo Elías Reynoso Ortega, Pamela Tejada-Tejada, Gustavo Gandini, Luis Enrique Rodríguez de Francisco, Ulises Javier Jáuregui-Haza

**Affiliations:** 1 Área de Ciencias Básicas y Ambientales, Instituto Tecnológico de Santo Domingo (INTEC), Avenida de Los Próceres #49, Los Jardines del Norte, Santo Domingo 10602, Dominican Republic; 1086710@est.intec.edu.do (M.E.R.O.); pamela.tejada@intec.edu.do (P.T.-T.); luis.defrancisco@intec.edu.do (L.E.R.d.F.); 2 Asociación BANELINO, Ave. Miguel Crespo, Frente a INPONOR, Mao 61000, Dominican Republic; gustavoarmandogandini@gmail.com

**Keywords:** *Sargassum*, liquid biofertilizer, anaerobic fermentation, trace metal distribution, soil–plant system

## Abstract

The recurrent influx of pelagic *Sargassum* spp. along Caribbean coastlines poses a significant environmental challenge while offering potential as a resource-recovery agricultural input. However, agricultural reuse of *Sargassum* biomass raises concerns regarding salinity and trace-metal distribution within the soil–plant–food continuum. This study evaluated the short-term elemental response to a *Sargassum*-Based Liquid Biofertilizer (SBLB) produced via controlled anaerobic fermentation, using tomato (*Solanum lycopersicum* L.) grown under greenhouse conditions. Raw biomass, fermented biofertilizer, irrigation water, soils, vegetative tissues, and fruits were chemically characterized. Elemental concentrations were quantified by ICP–OES and ICP-MS and treatment effects were analyzed using one-way and two-way ANOVA (*p* < 0.05). Anaerobic fermentation resulted in lower measured concentrations of sodium, arsenic, and selected trace elements in the liquid fraction relative to raw biomass. SBLB application increased soil macronutrient availability (N, P, K, Ca, Mg), while soil trace-metal concentrations remained within international reference ranges during the experimental period. Metals of concern (As, Cd, Pb, Ni, Cr) showed no detectable short-term enrichment in soils, vegetative tissues, or fruits relative to controls. In tomato fruits, arsenic, cadmium, and lead were below the limit of quantification across all treatments. Within the experimental timeframe, SBLB application was not associated with detectable trace-element accumulation in the soil–plant system. Long-term field studies and detailed soil physicochemical characterization are required to evaluate cumulative effects under repeated applications.

## 1. Introduction

The recurrent arrival of *Sargassum* spp. along Caribbean coastlines has become one of the most urgent environmental challenges of the past decade [1,2,3]. Significant accumulations of floating biomass disrupt tourism, fishing, and coastal ecosystems while incurring high management costs for local communities [4,5,6,7]. However, the high organic content and mineral richness of *Sargassum natans* and *Sargassum fluitans* also make these macroalgae promising feedstocks for bioresource recovery within circular bioeconomic frameworks [3,8,9,10,11]. Recent studies have shown that controlled bioconversion of *Sargassum* can produce bioproducts [11,12,13,14] suitable for soil amendment, biofertilizer formulation [9,15], compost [16,17] and plant biostimulation [18,19,20,21,22,23], provided that salinity and trace metal contents are adequately managed.

Despite this potential, recurrent *Sargassum* influxes are still largely managed as an environmental burden rather than as a bioresource, and most collected biomass remains underutilized or is disposed of without value recovery [12]. As a result, material that continuously enters coastal systems with significant environmental and economic impacts is rarely integrated into productive value chains [24].

From a resource-recovery perspective, the reuse of recurrent marine biomass aligns conceptually with circular bioeconomy principles emphasizing waste-to-resource conversion and nutrient recycling [25,26,27,28]. However, the present study does not evaluate circular economy performance metrics or system-level integration; rather, it focuses specifically on trace element distribution and environmental assessment following soil application.

In this context, the environmental compatibility of *Sargassum*-Based Liquid Biofertilizers critically depends on the behavior of trace metals throughout the soil–plant–food system. Accordingly, the present research evaluates a *Sargassum*-based liquid biofertilizer (SBLB) produced by anaerobic fermentation [29], with particular emphasis on trace metal distribution following soil application rather than on agronomic performance.

Tomato (*Solanum lycopersicum* L.) was selected as a model plant because of its widespread cultivation and relevance to food safety assessments, enabling the evaluation of potential trace-metal transfer from soil to edible tissues under controlled greenhouse conditions. The growing interest in replacing or complementing chemical fertilizers with organic and microbial formulations has led to the use of fermentation-derived products and marine biomass inputs as alternative nutrient sources. Despite the growing number of reports on seaweed extracts and biostimulants [19,20,21,23,30,31], comprehensive assessments addressing the environmental compatibility of *Sargassum*-based liquid biofertilizers, particularly regarding trace element distribution across the soil–plant–fruit continuum, remain limited.

Therefore, this study aims to evaluate the chemical composition and trace element distribution associated with a *Sargassum*-Based Liquid Biofertilizer (SBLB), and to compare its behavior with that of conventional chemical fertilization and an unfertilized control. The findings provide experimental evidence supporting the environmental assessment of Caribbean *Sargassum* spp. valorization within a resource-recovery context from a soil and food safety perspective.

## 2. Materials and Methods

### 2.1. Collection and Preparation of Sargassum Biomass

**Figure 1:**
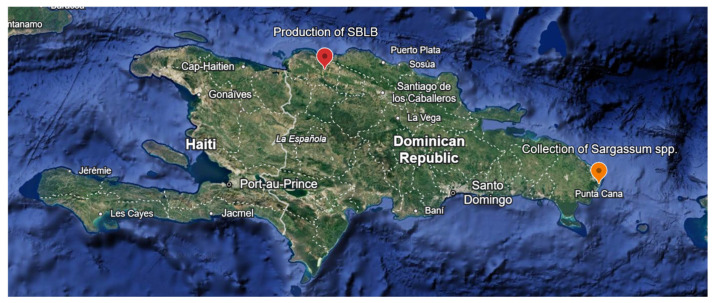
Location of *Sargassum* collection area (orange point) and Banelino Bio-ferments Plant (red point).

The collection of *Sargassum* spp. was conducted along the coast of La Altagracia Province (18°31′29″ N, 68°21′47″ W), Dominican Republic (Figure 1), between 20 and 100 m offshore, using artisanal fishing boats equipped with the LCM (Low-Cost Modular) collection system, as described by [32]. About one metric ton of freshly collected biomass, mainly consisting of *Sargassum natans* and *Sargassum fluitans*, was harvested during the summer of 2024.

The collected material was immediately washed offshore with seawater to remove sand, epiphytes, and debris, then drained and sun-dried in piles for five consecutive days until the moisture content was below 15%. After drying, the *Sargassum* was packed in cardboard boxes, labeled, and transported to the biopreparation plant of the Asociación BANELINO (Guayubin, Monte Cristi, Dominican Republic) for further processing.

For biomass characterization, approximately 100 g of dry material was collected from each box and combined to form a composite sample representative of the entire batch. The composite was thoroughly homogenized, ground to a particle size of less than 2 mm, and stored in polypropylene bags under dry, dark conditions until chemical analyses were conducted.

A composite sampling method was used without determining the percentages of each *Sargassum* morphotype arriving in the Caribbean. While this constitutes a limitation, it also reduces the cost of the *Sargassum* treatment process as a raw material for biofertilizer production.

### 2.2. Production of Sargassum-Based Liquid Biofertilizer

A *Sargassum*-Based Liquid Biofertilizer (SBLB) was produced in the Banelino Bio-ferments Plant, in *Hato del Medio Arriba*, Monte Cristi (19°41′38″ N, 71°18′56″ W). The exact location in the Dominican Republic is shown in Figure 1. The SBLB formulation was developed using sun-dried *Sargassum* biomass as the primary substrate at 8.75% (m/m), supplemented with molasses at 10.4% (*v*/*v*), whole milk at 2.2 (*v*/*v*), natural yogurt at 0.85% (*v*/*v*), and baker’s yeast (*Saccharomyces cerevisiae*) at 0.88%(m/m) as microbial and enzymatic activators, following the anaerobic fermentation procedure described by [15]. The biofermenter (a one-cubic-meter plastic cube, Figure S1, Supporting Information) was completed with fresh water to a total volume of 1 m^3^, sealed, and left to ferment under static, batch, oxygen-free conditions at ambient temperature (28 ± 2 °C), and an initial pH of 5.15 for 30 days. The process was considered complete when visible gas production ceased, indicating that the fermentation stage had stabilized under batch conditions. Approximately 900 L of biofertilizer were obtained and filtered through a 0.5 mm mesh and stored in high-density polyethylene (HDPE) containers at room temperature until use.

Physicochemical parameters such as pH and electrical conductivity were not monitored at the pilot-plant scale, as process optimization and monitoring had previously been conducted under controlled laboratory conditions [29]. In the present study, the focus was placed on post-fermentation chemical composition and environmental behavior following soil application.

### 2.3. Experimental Design and Planting

A commercial chemical fertilizer (N:P:K = 20:20:20) was included as a reference treatment to enable comparison of elemental inputs and trace metal behavior associated with conventional fertilization practices.

The experimental design comprised three fertilization treatments applied under controlled greenhouse conditions: (i) *Sargassum*-Based Liquid Biofertilizer (SBLB), (ii) commercial chemical fertilizer (N:P:K = 20:20:20), and (iii) an unfertilized control (no fertilizer input).

The greenhouse tomato (*Solanum lycopersicum* L.) experiment was conducted in the yard of the bioferment plant (Figure 1). Tomato seedlings were grown in trays for three weeks before transplanting.

Each treatment consisted of five replicate trays (n = 5), with four tomato seedlings per tray, for a total of 20 plants per treatment (total n = 60 plants). Trays were arranged following a completely randomized design within the greenhouse. The experimental design was specifically conceived to evaluate trace metal distribution and mobility within the soil–plant–fruit system, rather than agronomic performance or yield parameters.

For the SBLB treatment, applications were performed every 15 days throughout the 14-week cultivation period, alternating between foliar and soil applications with a one-week interval between each mode. Foliar applications (150 mL) were conducted at 5% (*v*/*v*) dilution, while soil applications consisted of 300 mL of undiluted SBLB per plant.

For the chemical fertilizer treatment, 57 g of a water-soluble N:P:K (20:20:20) formulation was applied per plant directly to the soil every 15 days, following the same schedule as the SBLB treatment. Applications were performed exclusively through soil irrigation. The unfertilized control received irrigation water only and followed the same irrigation schedule without nutrient input. Detailed information regarding application mode, dosage, frequency, and replication is provided in Supplementary Information (Table S1A).

### 2.4. Soil Sampling and Characterization

Soil sampling followed the FAO Good Agricultural Practices (GAP) methodology [33]. Subsequently, soil characterization was performed using the Global Soil Laboratory Network (GLOSOLAN) and Latin American Soil Laboratory Network (LATSOLAN) protocols to determine organic matter and nutrient contents.

Composite soil samples were collected before treatment application (initial soil) and after 14 weeks of cultivation (final soil) to evaluate changes in elemental composition and potential trace element accumulation associated with the fertilization treatments. Analyses included pH (1:2.5 H_2_O), electrical conductivity (EC), total nitrogen (Kjeldahl method), organic matter (Walkley–Black), available phosphorus (Bray II), and exchangeable potassium, calcium, and magnesium (ammonium acetate extraction).

All elemental analyses were performed by an external ISO/IEC 17025–accredited laboratory (AGQ Laboratories). Heavy metals (Cd, Cr, Cu, Fe, Mn, Ni, Pb, Zn) and metalloids, including arsenic (As), were quantified by inductively coupled plasma–mass spectrometry (ICP–MS) following microwave-assisted acid digestion according to USEPA Method 3050B [34], using the laboratory’s validated and accredited analytical protocols.

All measurements were conducted in duplicate and expressed on a dry-weight basis. Soil analyses were performed on composite samples to represent average treatment-level soil conditions rather than point-scale spatial variability, consistent with an environmental risk assessment approach.

### 2.5. Tissue and Fruit Sampling and Elemental Analysis

Plant sampling was designed to evaluate trace element mobility and potential accumulation within the soil–plant–fruit system under repeated applications of a *Sargassum*-Based Liquid Biofertilizer (SBLB), comparing chemical fertilization with an unfertilized control as the reference treatment. At the flowering stage, composite samples of stems and leaves were collected from each treatment to analyze elemental distribution in vegetative tissues. At harvest, additional composite samples of stems and leaves were gathered to examine changes in tissue elemental content over time between phenological stages. Tomato fruits were harvested to measure elemental concentrations in the edible tissues and evaluate food safety.

All plant tissues (stems, leaves, and fruits) were analyzed for macronutrients and trace elements of environmental concern (N, P, K, Ca, Mg, Na, Fe, Mn, Zn, Cu, As, Cd, Pb). Samples were prepared and quantified by ICP–OES following acid digestion, according to AOAC protocols [35]. Results are reported stems/leaves at flowering and harvest and for fruits.

### 2.6. Statistical Analysis

The mean and standard deviation were used to assess variability of the variables under study. The field test data were subjected to one-way ANOVA (type of treatment) or two-way ANOVA (type of treatment and vegetative state) to assess metal contents in stems and leaves, followed by Tukey’s mean comparison test (*p* < 0.05) when applicable. The concentrations of the chemical elements under study were analyzed as dependent variables. EXCEL and SPSS (version 21) were used for statistical analysis. In all cases, three samples from each treatment were randomly selected for analysis.

## 3. Results and Discussion

### 3.1. Characterization of Agricultural Inputs

The chemical composition of the agricultural inputs used in this study is presented in Table 1. The *Sargassum* biomass exhibited relatively high concentrations of potassium (36,267 mg·kg^−1^), calcium (>50,000 mg·kg^−1^), magnesium (10,071 mg·kg^−1^), and sodium (11,192 mg·kg^−1^), comparable to ranges previously reported for Caribbean *Sargassum* spp. [36,37,38]. These macronutrient levels are consistent with previous reports on *Sargassum*-derived soil amendments and fermented macroalgal products, in which elevated K, Ca, and Mg concentrations have been associated with agronomic relevance. However, elevated Na in raw biomass may pose salinity and sodicity concerns under repeated applications, particularly in salt-sensitive crops or poorly drained soils, and should therefore be interpreted with consideration of application rate, dilution, soil texture, and drainage conditions.

Regarding trace metals, the concentrations of mercury (Hg), cadmium (Cd), chromium (Cr), and lead (Pb) in both the raw *Sargassum* and the derived biofertilizer SBLB (Table 1), remained below the limit of quantification (<0.5 mg·kg^−1^) in most cases and were significantly lower than reference thresholds reported for agricultural matrices [47,49,51,55]. Notably, arsenic (As) concentration in the raw *Sargassum* (72.4 mg·kg^−1^) exceeds the reference range for use in agricultural soils (15–50 mg/kg). However, the arsenic content in SBLB is below the limit of quantification of the analytical method (<0.5 mg·kg^−1^). The lower measured arsenic concentrations in the liquid fraction relative to raw biomass are attributable to matrix partitioning (solid vs. liquid phases) and process-related dilution, rather than demonstrated chemical reduction or speciation transformation.

Additional insight into the fate of arsenic during anaerobic fermentation is provided by the chemical characterization of the solid residue remaining after fermentation (Table S2). While arsenic concentration in the liquid SBLB was below the limit of quantification, the solid residue retained a measurable arsenic concentration (8 mg·kg^−1^), substantially lower than that of the raw *Sargassum* biomass (72.4 mg·kg^−1^). These results indicate partitioning of arsenic between the solid and liquid fractions during fermentation, as well as dilution effects associated with process conditions. No arsenic speciation analysis, dissolved fraction assessment, or redox-controlled geochemical mechanisms were evaluated in this study. Because regulatory frameworks typically consider total arsenic, cumulative loading rates, and application frequency, these results should be interpreted cautiously. Long-term field monitoring, arsenic speciation analysis, and soil physicochemical characterization are required to assess cumulative risks under repeated applications and under site-specific background geochemistry.

The chemical fertilizer (N: P: K = 20:20:20) exhibited elevated iron (Fe, 5299 mg·kg^−1^) and manganese (Mn, 158 mg·kg^−1^) concentrations, consistent with its inorganic composition, while the SBLB displayed markedly lower levels of these elements (Fe 25.4 mg·kg^−1^; Mn 2 mg·kg^−1^). These differences reflect the contrasting origins of nutrients in mineral fertilizers versus fermentation-derived organic amendments.

Concerning proximate composition, the raw *Sargassum* contained 54 g·100 g^−1^ carbohydrates, 6 g·100 g^−1^ proteins, and 0.43 g·100 g^−1^ lipids (Table 1), consistent with the biochemical signature of brown algae dominated by polysaccharides such as alginate, mannitol, and laminarin [60,61]. After fermentation, the SBLB-INTEC showed lower carbohydrate and protein content, reflecting microbial consumption of available substrates during anaerobic metabolism.

The pH and EC values of SBLB indicate mild acidity and moderate ionic strength (pH 4.98; EC 762 µS·cm^−1^), within ranges commonly reported as manageable for foliar or soil application when appropriately diluted [62]. Overall, the input characterization indicates that measured trace element concentrations in the liquid SBLB were lower than in raw biomass. Nevertheless, the long-term implications of sodium accumulation and arsenic loading remain dependent on site-specific soil conditions, background geochemistry, and management practices.

### 3.2. Soil Elemental Response

The elemental composition of soil before and after treatment application is presented in Table 2 to evaluate trace metal accumulation and nutrient shifts relevant to ecological safety within the 14-week greenhouse timeframe.

**Table 2:** Soil macronutrients, micronutrients, heavy metals, and metalloids concentrations (mg·kg^−1^) before (initial) and after (final) application of fertilization treatments.

Element	Initial Soil	Final Soil			Reference Range
		Chemical Fertilizer	SBLB	Control	
**Macronutrients and micronutrients**					
Phosphorus (P)	209	1308 ± 118	974 ± 88	1065 ± 96	–
Nitrogen (N)	695	2355	1933	2127	–
Potassium (K)	868	3449 ± 241	2918 ± 204	3221 ± 225	–
Calcium (Ca)	6312 ± 379	9327 ± 560	10,190 ± 611	16,347 ± 981	–
Magnesium (Mg)	5971	7235 ± 290	6992 ± 280	7050 ± 282	–
Sodium (Na)	174	319 ± 19	299 ± 18	341 ± 21	–
Iron (Fe)	19,568	19,745 ± 790	19,658 ± 786	20,505 ± 820	–
Manganese (Mn)	326	345 ± 24.2	330 ± 23.1	340 ± 23.8	–
Copper (Cu)	28.3 ± 3.4	38.3 ± 4.6	35.5 ± 4.3	37.0 ± 4.4	60–200
Zinc (Zn)	32.6	53.9 ± 4.9	49.0 ± 4.4	51.2 ± 4.6	70–600
Cobalt (Co)	12.3 ± 0.61	11.0 ± 0.55	10.9 ± 0.54	11.3 ± 0.57	25–50
Molybdenum (Mo)	0.237	0.334 ± 0.03	0.285 ± 0.026	0.358 ± 0.032	10
**Heavy metals and metalloids**					
Arsenic (As)	2.73 ± 0.27	2.93 ± 0.29	2.84 ± 0.28	3.97 ± 0.4	15–50
Cadmium (Cd)	0.054 ± 0.0032	0.0590 ± 0.0035	0.0529 ± 0.0032	0.0585 ± 0.0035	1–37
Chromium (Cr)	68.3 ± 4.8	63.5 ± 4.4	63.7 ± 4.5	71.1 ± 5.0	50–280
Nickel (Ni)	44.4 ± 3.6	41.8 ± 3.4	41.8 ± 3.3	44.1 ± 3.5	100–139
Lead (Pb)	2.04 ± 0.33	1.66 ± 0.26	1.68 ± 0.27	1.68 ± 0.27	100–1000

Note: Final soil values correspond to measurements after 14 weeks of treatment application. Values are reported as mean ± analytical uncertainty provided by the analytical laboratory. Uncertainty does not represent biological variability. Pb concentrations reflect the natural geochemical background of the experimental site and did not increase after SBLB application. The LOQ for each element is provided in the Supplementary Material (Table S3). Reference range [46,47,48,49,50,51,52,53,54,55,56,57,58,59].

Following the 14-week cultivation period, soils treated with SBLB exhibited increases in nitrogen (N), phosphorus (P), and potassium (K) to 1933; 974; and 2918 mg·kg^−1^, respectively. These values were comparable to those obtained with the chemical fertilizer (N: 2355, P: 1318, and K: 3449 mg·kg^−1^). The enrichment in N, P, and K reflects the mineralization of organic compounds during fermentation and the gradual release of nutrients into the rhizosphere.

Table 3 presents the results of the one-way analysis of variance, considering soil composition before and after as the independent variable for the different treatments (See Supporting information file Statistical Analysis for soil: SI-SA-Soil). Of all the evaluated elements, statistically significant variations were observed in only eight: Ca, K, P, Na, As, Mo, Zn, and Mg. The increase in macronutrients (Ca, K, P, and Na) is explained by their presence in the chemical fertilizer, the liquid biofertilizer from *Sargassum*, and the irrigation water. Regarding heavy metals, arsenic (As) and molybdenum (Mo) showed significant increases—relative to the initial soil composition—only in the control treatment, whereas Zn increased in both the chemically fertilized soil and the control. It is important to note that, in all cases, the concentrations of heavy metals after the different treatments—including the control treatment—remained below the maximum allowable limits established in agricultural soil standards. These findings suggest that, within the 14-week experimental period, the use of liquid biofertilizer improves soil composition, and no treatment-specific increases in regulated heavy metals were observed relative to reference ranges.

**Table 3:** Statistical analysis of the influence of different treatments on soil composition.

	ANOVA		Tukey’s Mean Comparison (*p*-Values)					
Variable	F	*p* -Value	IS vs. FS-CF	IS vs. FS-SBLB	IS vs. FS-Control	FS-CF vs. FS-SBLB	FS-CF vs. FS-Control	FS-SBLB vs. FS-Control
Ca	118.67	<0.0001	0.0025	0.0005	*p* < 0.0001	0.4403	*p* < 0.0001	*p* < 0.0001
K	35.28	0.0003	0.0002	0.0009	0.0004	0.0964	0.6231	0.4178
P	29.75	0.0005	0.0003	0.0025	0.0014	0.0264	0.0932	0.7034
Na	20.04	0.0016	0.0024	0.0051	0.0011	0.6013	0.5338	0.1227
As	10.01	0.0044	0.8619	0.9719	0.0057	0.9841	0.0155	0.0098
Mo	5.92	0.0318	0.1026	0.5362	0.0447	0.2725	0.7562	0.0823
Zn	5.47	0.0375	0.028	0.0799	0.0493	0.5982	0.8885	0.9343
Mg	5.08	0.0438	0.0321	0.0752	0.0611	0.7304	0.8533	0.9939
Co	3.96	0.053						
Cu	3.39	0.0742						
Cd	2.64	0.1214						
Cr	1.91	0.2062						
Pb	1.36	0.3228						
Fe	0.76	0.558						
Ni	0.51	0.6879						
Mn	0.29	0.8317						

Note: In gray, *p*-values are lower than 0.05. IS: Initial soil; FS-CF: Final soil treated with chemical fertilizer; FS-SBLB: Final soil treated with *Sargassum*-based liquid biofertilizer; FS-Control: Final soil untreated control.

Notably, the SBLB treatment showed a moderate but consistent improvement in calcium (Ca) and magnesium (Mg) (10,190 and 6992 mg·kg^−1^, respectively), exceeding those observed under chemical fertilizer and control treatments. Although exchangeable fractions were not determined in this research, Ca and Mg are recognized as essential divalent cations involved in soil structural stability and plant physiological processes [63].

The sodium (Na) content in SBLB (299 mg·kg^−1^) remained low and close to baseline levels (174 mg·kg^−1^), indicating no evidence of short-term salinity buildup despite the marine origin of the biomass. This finding is consistent with previous evidence showing that fermentation ensures the reduction in soluble Na concentrations through phase partitioning and process-related dilution [29].

All measured heavy metals in post-harvest soil were below the recommended limits for agricultural soils [64,65]. Arsenic (As) levels ranged from 2.73 to 3.97 mg·kg^−1^ across treatments, far below the maximum permissible level (15–50 mg·kg^−1^). Cadmium (Cd) and chromium (Cr) concentrations in SBLB and amended soils were 0.0529 ± 0.0032 and 63.7 ± 4.5 mg·kg^−1^ (Table 2), respectively, indicating values within the natural range for tropical mineral soils and comparable to the control. Crucially, no increase in Pb levels was observed after SBLB application, suggesting no detectable short-term Pb enrichment attributable to SBLB under the tested greenhouse conditions.

Copper (Cu), zinc (Zn), and iron (Fe) levels in the SBLB treatment (35.5, 49, and 19,658 mg·kg^−1^, respectively) were comparable to the organic and inorganic references, reflecting that micronutrient enrichment derived mainly from soil background rather than fertilizer input. Cobalt (Co) and molybdenum (Mo) concentrations remained low (≈10–11 mg·kg^−1^ and 0.28 mg·kg^−1^, respectively), consistent with FAO recommended limits for agricultural soils.

Soils under SBLB showed increased macronutrients relative to the initial condition, while trace metals (e.g., As, Cd, Cr, Ni) remained within the reported reference ranges for agricultural soils.

Overall, soils treated with SBLB showed improved macronutrient availability relative to the initial soil condition, while trace metals (e.g., As, Cd, Cr, Ni) remained within reported reference ranges for agricultural soils. Although Pb concentrations were elevated across all treatments due to local geochemical background, no additional Pb input was attributable to SBLB application.

### 3.3. Elemental Distribution in Vegetative Tissues

The elemental composition of tomato stems and leaves at the flowering and harvest stages is shown in Table 4, enabling evaluation of internal nutrient redistribution and potential accumulation of trace metals during plant development.

**Table 4:** Elemental composition of stems and leaves of tomato plants at flowering and harvest stages under different fertilization treatments (mg·kg^−1^).

Element	Stage	Chemical Fertilizer	SBLB	Control	Limits in Vegetables
Arsenic (As)	Flowering	0.51 ± 0.07	0.55 ± 0.07	0.46 ± 0.06	–
	Harvest	0.38 ± 0.05	0.39 ± 0.05	0.40 ± 0.05	
Cadmium (Cd)	Flowering	0.113 ± 0.015	0.108 ± 0.014	0.097 ± 0.013	0.2
	Harvest	0.070 ± 0.0091	0.066 ± 0.0086	0.060 ± 0.0084	
Chromium (Cr)	Flowering	3.40 ± 0.41	3.40 ± 0.4	3.20 ± 0.38	–
	Harvest	5.00 ± 0.60	4.40 ± 0.53	4.60 ± 0.56	
Copper (Cu)	Flowering	12.0 ± 1.6	13.0 ± 1.7	11.0 ± 1.5	73.3
	Harvest	6.70 ± 0.87	7.40 ± 0.96	6.60 ± 0.85	
Iron (Fe)	Flowering	994 ± 89	906 ± 82	951 ± 86	425.5
	Harvest	546 ± 49	583 ± 52	539 ± 49	
Manganese (Mn)	Flowering	69.0 ± 3.5	60.9 ± 3.0	73.4 ± 3.7	–
	Harvest	55.1 ± 2.8	49.4 ± 2.5	41.8 ± 2.1	
Lead (Pb)	Flowering	0.716 ± 0.086	0.401 ± 0.048	0.357 ± 0.043	0.3
	Harvest	0.350 ± 0.042	0.332 ± 0.04	0.290 ± 0.034	
Zinc (Zn)	Flowering	39.0 ± 5.0	46.0 ± 6.0	45.0 ± 5.8	99.4
	Harvest	63.0 ± 8.2	96.0 ± 13	57.0 ± 7.4	
Phosphorus (P) *	Flowering	0.342 ± 0.044	0.296 ± 0.039	0.476 ± 0.062	–
	Harvest	0.247 ± 0.032	0.192 ± 0.025	0.168 ± 0.022	
Nitrogen (N) *	Flowering	3.60	3.30	2.60	–
	Harvest	2.70	2.40	2.40	
Potassium (K) *	Flowering	4.75 ± 1.00	4.47 ± 0.94	5.95 ± 1.3	–
	Harvest	3.70 ± 0.78	3.65 ± 0.77	2.88 ± 0.6	
Calcium (Ca) *	Flowering	2.01 ± 0.16	2.26 ± 0.18	1.89 ± 0.15	–
	Harvest	2.40 ± 0.19	2.12 ± 0.17	2.02 ± 0.16	
Magnesium (Mg) *	Flowering	0.691 ± 0.069	0.691 ± 0.069	0.924 ± 0.092	–
	Harvest	0.766 ± 0.077	0.701 ± 0.070	0.68 ± 0.068	
Sodium (Na)	Flowering	5661	5852	6158	–
	Harvest	4996	6785	7223	

Note: * Values expressed as %, while all other elements are reported as mg·kg^−1^. Values are reported as mean ± analytical uncertainty provided by the analytical laboratory. Uncertainty does not represent biological variability. The LOQ for each element is provided in the Supplementary Material (Table S4). Limits on vegetables were taken from [52].

Across both phenological stages, macronutrient concentrations followed expected physiological trends, with moderate decreases from flowering to harvest reflecting nutrient remobilization toward reproductive organs. Nitrogen, phosphorus, potassium, calcium, and magnesium levels in SBLB-treated plants were comparable to those observed under chemical fertilization and remained within typical ranges reported for tomato vegetative tissues.

Table 5 presents the results of the statistical analysis comparing the influence of two independent variables—the type of treatment (Chemical fertilizer, SBLB and control) and the vegetative stage of the plant (flowering versus harvest)—on the concentrations of twelve elements measured in tomato leaf and stem tissues (see Supporting Information, SI-SA-Vegetative State). The type of treatment had a significant effect on Pb, Zn, Mn, P, and Ca; the vegetative stage affected all elements except Ca and Mg; and the interaction between the two independent variables was significant for the concentrations of Pb, Zn, Mn, P, and Mg in the leaf and stem tissues.

In the case of lead (Pb), it was observed that, across all treatments, its concentration in leaf and stem tissues was higher than the general reference value reported for vegetables in the FAO/WHO Codex Alimentarius (CXS 193-1995) [52], with levels doubling when chemical fertilizer was applied during the flowering stage.

However, as discussed in Section 3.4, this elevation in vegetative tissues did not translate into increased Pb concentrations in harvested tomato fruits, which remained below the limit of quantification across all treatments. All other elements remained within the general reference values reported for vegetables, with a consistent pattern of higher concentrations in leaf and stem tissues during the flowering stage than during the fruiting stage.

Trace and heavy metal concentrations in stems and leaves remained low throughout the cultivation cycle. Cadmium, arsenic, and lead values under SBLB treatment were comparable to or lower than those observed under chemical fertilization and the control, with no evidence of progressive accumulation from flowering to harvest. Although iron and zinc concentrations increased at harvest, particularly in SBLB-treated plants, values remained within ranges commonly reported for tomato foliage and did not translate into increased accumulation in fruits, as will be shown later.

Overall, no consistent enrichment patterns attributable to SBLB application were observed in vegetative tissues during the 14-week experimental period. Under the tested greenhouse conditions, SBLB application did not promote progressive accumulation of potentially toxic elements in leaf and stem tissues.

### 3.4. Elemental Composition and Food Safety of Fruits

The elemental composition of tomato fruits is shown in Table 6 to evaluate the potential buildup of nutrients and trace metals in edible tissues under various fertilization treatments.

Table 7 presents the results of the one-way analysis of variance, where the independent variable is the type of treatment and the dependent variables are the concentrations of various elements in tomato fruits (See Supporting information file Statistical Analysis for element content in fruits: SI-AS-Fruits). For this analysis, only seven of the fifteen measured elements were considered. The macronutrients P, N, K, and Ca were excluded due to insufficient replicates for statistical testing. Likewise, Na, As, Cd, and Pb were omitted because their concentrations remained below the quantification limit of the analytical method. Among the seven elements analyzed, six (Ni, Cu, Fe, Mg, Cr, and Zn) showed statistically significant differences among treatments, with all exhibiting higher concentrations in fruits treated with chemical fertilizer than in those treated with SBLB. However, in all cases, the levels of these micronutrients and heavy metals were below the limits established for human consumption, reaffirming the suitability of SBLB for use in organic agriculture. A more detailed analysis for each group of elements is presented below.

#### 3.4.1. Macronutrients

Tomato fruits fertilized with SBLB showed potassium (K) levels of 3752 mg·kg^−1^, slightly higher than those with chemical fertilizer (3653 mg·kg^−1^) and higher than the unfertilized control (Table 6). Nitrogen (N), phosphorus (P), calcium (Ca), and magnesium (Mg) concentrations in the SBLB treatment were lower than those in chemically fertilized fruits, but remained within physiological ranges reported for healthy tomato [66,67,68]. These results indicate that SBLB supplied essential nutrients without promoting excessive accumulation in edible tissues.

Overall, the macronutrient distribution (high K, moderate N and P, adequate Ca and Mg) reflects a balanced nutrient availability consistent with the slow-release behavior of organically stabilized biofertilizers [69].

#### 3.4.2. Trace Metals in Fruits

Heavy metal concentrations in tomato fruits were below international food safety limits [52]. Arsenic (As), cadmium (Cd), and lead (Pb) were below the limit of quantification (<0.01 mg·kg^−1^) across all treatments (Table 6), indicating no detectable accumulation in edible tissues under the 14-week greenhouse conditions tested. Copper (Cu), zinc (Zn), and iron (Fe) concentrations were slightly lower in SBLB-treated fruits (0.19, 0.76, and 2.4 mg·kg^−1^, respectively) than in those receiving chemical fertilizer, without evidence of enhanced transfer to edible tissues relative to the inorganic fertilizer treatment. This behavior is consistent with previous reports on seaweed-based fertilizers, in which organic ligands, such as alginates and phenolic compounds, can chelate metal ions, thereby reducing their bioavailability [70]. Nickel (Ni) levels remained low, and no short-term enrichment of potentially toxic elements was observed during the experimental period. These findings provide preliminary data for future dietary risk assessments associated with SBLB application, similar to other studies in Romania that investigated the bioaccumulation and soil–plant transfer of trace metals in edible crops [71].

### 3.5. Environmental Implications

The overall soil chemical response indicates that the application of the *Sargassum*-Based Liquid Biofertilizer was not associated with detectable short-term soil contamination or salinity increases within the 14-week greenhouse experiment, while replenishing key macronutrients and secondary cations essential for soil fertility. The stability of trace metal concentrations in soils treated with SBLB is consistent with the measured elemental concentrations in the fermented liquid; however, no direct assessment of metal speciation, fractionation, or bioavailability was performed in this research. These findings align with recent studies on *Sargassum* digestates produced via anaerobic digestion, which, despite containing trace elements such as Zn and As, significantly enhance biomass in tomato seedlings when incorporated into circular economy strategies [72]. Lower measured arsenic and sodium concentrations in the fermented liquid compared with raw biomass, combined with the absence of trace-element enrichment in soils, vegetative tissues, and fruits, indicate no detectable short-term accumulation across the soil–plant–fruit continuum under the experimental conditions tested.

It is important to note that part of the observed variability in soil elemental concentrations, particularly for sodium, calcium, magnesium, and selected micronutrients, can be attributed to the chemical composition of the irrigation water used during the experiment (Table 1). Recent research has highlighted that the interaction of trace metals within the water–soil–plant continuum is a critical factor for food safety, as the chemical quality of irrigation water directly influences metal sorption onto the soil and subsequent plant uptake [73]. Irrigation water had moderate electrical conductivity (1274 µS·cm^−1^) and measurable concentrations of Ca, Mg, Na, Fe, and Zn, likely contributing to background nutrient inputs independent of fertilization treatments. However, trace metals of environmental concern (As, Cd, Cr, Ni, Pb) in irrigation water were present at very low concentrations, well below guideline values, indicating that irrigation did not represent a contamination source within this experimental framework.

The convergence of evidence from soil, plant tissues, fruits, and irrigation water suggests that the observed elemental variations were associated with controlled nutrient inputs rather than detectable short-term trace element accumulation.

These findings are consistent with previous studies on *Sargassum*-derived fermentation-derived liquid amendments (termed “biofertilizers” in the agronomic literature) applied to banana cultivation, which showed improvements in soil nutrient status without deterioration of soil chemical quality or trace metal accumulation [29]. While the results support short-term environmental compatibility under greenhouse conditions, long-term field evaluations, repeated application studies, and detailed soil physicochemical characterization are necessary to fully assess cumulative effects, contaminant transport dynamics, and potential changes in metal bioavailability.

## 4. Conclusions

This study evaluated the short-term distribution of trace elements across the soil–plant–fruit continuum following the application of a *Sargassum*-based liquid biofertilizer (SBLB) produced via controlled anaerobic fermentation under greenhouse conditions. The fermented product (SBLB) contained very low concentrations of priority metals, including arsenic—remaining below the analytical quantification limit—despite the high arsenic content in the raw biomass. These results indicate a measurable difference between the solid biomass and the liquid fraction obtained after fermentation, without assessing speciation or long-term mobility.

Under greenhouse conditions, arsenic, cadmium, and lead remained below the limit of quantification in tomato fruits across fertilization treatments, indicating no detectable short-term accumulation in edible tissues at the tested application rates. Vegetative tissues likewise showed no consistent short-term enrichment patterns attributable to SBLB during the 14-week experimental period. At the soil level, SBLB application increased macronutrient content relative to baseline values, while maintaining trace metals (As, Cd, Cr, Ni) within reported reference ranges. These observations were statistically validated using one-way ANOVA (treatment type) and two-way ANOVA (treatment × vegetative stage for stem and leaf metal content), followed by Tukey’s multiple comparison test (*p* < 0.05) where appropriate.

Overall, the findings indicate that, under the controlled greenhouse conditions and within the 14-week timeframe evaluated, SBLB application was not associated with detectable short-term trace-element enrichment in soils, vegetative tissues, or fruits. Future research should confirm these trends under field conditions and, critically, incorporate long-term (multi-season) monitoring and standardized risk indicators—such as bioaccumulation and transfer factors—to strengthen environmental risk assessments.

## Figures and Tables

**Table 1:** Physicochemical, biomolecular, and elemental profile of raw *Sargassum*, Irrigation water, chemical fertilizer, and the *Sargassum*-Based Liquid Biofertilizer (SBLB).

Parameter	Unit	*Sargassum*	Irrigation Water	Chemical Fertilizer	SBLB	Reference Range
**Alkali and alkaline earth metals**						
Barium (Ba)	mg·kg^−1^	30.0	n.d.	n.d.	<0.12	—
Beryllium (Be	mg·kg^−1^	<0.05	n.d.	n.d.	<0.05	—
Potassium (K)	mg·kg^−1^	36,267	n.d.	>50,000	5996	3200 †
Calcium (Ca)	mg·kg^−1^	>50,000	93.0 ± 15.0 *	27,986	2042	—
Magnesium (Mg)	mg·kg^−1^	10,071	43.3 ± 5.6 *	4815	762	628 †
Sodium (Na)	mg·kg^−1^	11,192	135 ± 18 *	3714	599	948 †
**Heavy metals and metalloids**						
Mercury (Hg)	mg·kg^−1^	<0.5	n.d.	n.d.	<0.5	0.5 ‡
Thallium (Tl)	mg·kg^−1^	<15.0	n.d.	n.d.	<15.0	—
Vanadium (V)	mg·kg^−1^	5.00	n.d.	n.d.	<0.3	—
Aluminum (Al)	mg·kg^−1^	33.0	n.d.	n.d.	6.00	—
Arsenic (As)	mg·kg^−1^	72.4	0.0030 ± 0.00036 *	5.00	<0.5	15–50 ‡
Nickel (Ni)	mg·kg^−1^	3.00	<0.001 *	6.00	<0.6	100–139 ‡
Chromium (Cr)	mg·kg^−1^	1.00	<0.001 *	69.0	<0.3	50–280 ‡
Cadmium (Cd)	mg·kg^−1^	1.00	<0.0002 *	2.00	<0.2	1–37 ‡
Copper (Cu)	mg·kg^−1^	8.00	0.015 ± 0.0023 *	8.00	4.00	60–200 ‡
Iron (Fe)	mg·kg^−1^	82.2	0.191 ± 0.025 *	5299	25.4	—
Manganese (Mn)	mg·kg^−1^	24.0	0.0094 ± 0.0015 *	158	2.00	—
Zinc (Zn)	mg·kg^−1^	7.44	0.050 ± 0.0054 *	57.6	<3.0	70–600 ‡
Cobalt (Co)	mg·kg^−1^	<0.7	n.d	0.90	<0.7	25–50 ‡
Molybdenum (Mo)	mg·kg^−1^	<1.2	0.0020 ± 0.0002 *	6.30	—	—
Lead (Pb)	mg·kg^−1^	<1.5	0.0021 ± 0.00023 *	<1.5	<1.5	100–1000 ‡
**Biomolecules and physicochemical properties**						
Total carbohydrates	g·100 g^−1^	54.0	n.d.	n.d.	0.73	—
Total proteins	g·100 g^−1^	6.00	n.d.	n.d.	1.00	—
Total lipids	g·100 g^−1^	0.43	n.d.	n.d.	0.20	—
pH	—	n.d.	7.66 *	5.39	4.98	—
Electrical conductivity	µS·cm^−1^	n.d.	1274 ± 83 *	>30,000	3654	—
Salinity	ppt	n.d.	n.d.	n.d.	15.9	—

Note: † Reference values found in other liquid biofertilizer [39,40,41,42,43,44,45]; ‡ Values permissible limits in [46,47,48,49,50,51,52,53,54,55,56,57,58,59]. n.d. Not determined; * Concentrations expressed in mg·L^−1^. Values reported as “<” indicate concentrations below the limit of quantification (LOQ) of the analytical method (Table S1).

**Table 5:** Two-way ANOVA evaluating the effects of fertilization treatment (Chemical fertilizer SBLB and control) and vegetative state (flowering vs. harvest) on elemental concentrations in tomato stems and leaves.

	ANOVA						Tukey’s Mean Comparison (Number of Significant Interactions)		
Variable	F_Treat	p_Treat	F_Stage	p_Stage	F_Treat×Stage	p_Treat×Stage	Treat × Stage Significant Interactions	Significant Comparisons (Flowering)	Significant Comparisons (Harvest)
Pb	27.46	<0.0001	47.17	<0.0001	16.54	0.0004	5	2	0
Zn	12.45	0.0012	57.54	<0.0001	8.81	0.0044	6	0	2
Mn	8.24	0.0056	182.39	<0.0001	20.30	0.0001	10	1	2
P	5.97	0.0158	81.74	<0.0001	13.84	0.0008	8	2	1
Ca	4.14	0.043	2.53	0.1376	3.69	0.0562	1	0	0
Mg	3.04	0.0857	2.19	0.1648	7.06	0.0094	4	2	0
Cd	1.92	0.1891	54.63	<0.0001	0.11	0.8934	7	0	0
Cu	1.77	0.2115	69.52	<0.0001	0.35	0.7133	9	0	0
Cr	0.76	0.4903	33.64	<0.0001	0.59	0.5703	4	0	0
As	0.7	0.515	17.54	0.0012	1.13	0.3547	1	0	0
Fe	0.26	0.776	142.09	<0.0001	1.26	0.3183	9	0	0
K	0.22	0.8047	14.26	0.0026	2.69	0.1086	1	0	0

Note: In gray, *p*-values < 0.05 were considered statistically significant. Treat: fertilization treatment (Chemical fertilizer, SBLB, Control); Stage: vegetative stage (flowering F, harvest H). Details of Tukey post hoc comparisons are provided in the Supporting Information (SI-SA-Vegetative State).

**Table 6:** Elemental composition of harvested tomato fruits under different fertilization treatments and international reference limits (mg·kg^−1^).

Parameter	Chemical Fertilizer	SBLB	Control	Limits in Vegetables
**Macronutrients and micronutrients**				
Phosphorus (P) *	22	9.1	8.6	–
Nitrogen (N)	149	72	77	–
Potassium (K) *	3653	3752	3148	–
Calcium (Ca) *	27	20	19	–
Magnesium (Mg)	116.00 ± 5.8	91.0 ± 4.5	81 ± 4.0	–
Sodium (Na) *	<125	<125	126	–
Chromium (Cr)	0.0749 ± 0.0048	0.0651 ± 0.0042	0.0957 ± 0.0061	–
Copper (Cu)	0.2452 ± 0.0056	0.1884 ± 0.0043	0.1587 ± 0.0036	73.3
Iron (Fe)	3.10 ± 0.088	2.40 ± 0.068	3.90 ± 0.11	425.5
Manganese (Mn)	0.50 ± 0.1	0.356 ± 0.072	0.343 ± 0.069	–
Zinc (Zn)	1.54 ± 0.26	0.759 ± 0.130	0.653 ± 0.110	99.4
**Heavy metals and metalloids**				
Arsenic (As)	<0.01	<0.01	<0.01	–
Cadmium (Cd)	<0.01	<0.01	<0.01	0.2
Lead (Pb)	< 0.01	< 0.01	< 0.01	0.3
Nickel (Ni)	0.072 ± 0.0018	<0.01	0.019 ± 0.00048	67.9

Note: * Values expressed as mg·100 g^−1^, while all other elements are reported as mg·kg^−1^. Values are reported as mean ± analytical uncertainty provided by the analytical laboratory. Uncertainty does not represent biological variability. Values reported as “<” indicate concentrations below the limit of quantification (LOQ) of the analytical method. The LOQ for each element is provided in the Supplementary Material (Table SI.5). Limits on vegetables were taken from [52].

**Table 7:** Statistical analysis of the influence of different treatments on the element content in tomato fruits.

	ANOVA		Tukey’s Mean Comparison (*p*-Values)		
Variable	F	*p* -Valor	CF vs. SBLB	CF vs. Control	SBLB vs. Control
Ni	1525.39	<0.0001	<0.0001	<0.0001	0.009
Cu	276.29	<0.0001	<0.0001	<0.0001	0.0005
Fe	207.21	<0.0001	0.0002	0.0001	<0.0001
Mg	41.85	0.0003	0.0017	0.0003	0.0973
Cr	28.21	0.0009	0.1227	0.0059	0.0008
Zn	21.86	0.0018	0.0043	0.0022	0.7594
Mn	3.42	0.1019			

Note: In gray, *p*-values are lower than 0.05. CF: Fruits treated with chemical fertilizer; SBLB: Fruits treated with *Sargassum*-based liquid biofertilizer; Control: Fruits of untreated control.

## Data Availability

The original contributions presented in this study are included in the article/Supplementary Material. Further inquiries can be directed to the corresponding author.

## Supplementary Materials

The following supporting information can be downloaded at: https://www.mdpi.com/article/10.3390/plants15060901/s1, Table S1: The limit of quantification in biofertilizer; Table S1A. Summary of fertilization treatments applied during the 14-weeks greenhouse experiment; Table S2: Elemental composition of raw Sargassum biomass and solid residue obtained after anaerobic fermentation; Table S3: The limit of quantification in soils; Table S4: The limit of quantification in vegetative tissues; Table S5: The limit of quantification in tomato fruits; Figure S1: One-cubic-meter cube fermeter at Banelino Bio-ferments Plant, in Hato del Medio Arriba, Monte Cristi, Dominican Republic.
