# Supplementary material for: Short-Term Trace Element Distribution Following Application of Sargassum-Based Liquid Biofertilizer in a Soil–Plant–Tomato Fruit System
Source: Plants (Basel). 2026 Mar 14;15(6):901. doi: 10.3390/plants15060901 (PMC13030466; doi:10.3390/plants15060901)
Supplement: Supplementary file 1 [file plants-15-00901-s001.zip › plants-4121369-supplementary.pdf]

# Supporting Information

# Short-Term Trace Element Distribution Following Application of Sargassum-Based Liquid Biofertilizer in a Soil–Plant–Tomato Fruit System.

**Table SI.1:** The limit of quantification in biofertilizer.

| Element | The limit of quantification | Method |
|---|---|---|
| **Alkali and alkaline earth metals** | | |
| Barium (Ba) | 0,12 mg/kg | PP-281 |
| Beryllium (Be | 0,05 mg/kg | PP-281 |
| Potassium (K) | 85,0 mg/kg | PP-281 |
| Calcium (Ca) | 33,0 mg/kg | PP-281 |
| Magnesium (Mg) | 31,0 mg/kg | PP-281 |
| Sodium (Na) | 26,0 mg/kg | PP-281 |
| **Heavy metals and metalloids** | | |
| Mercury (Hg) | 0,500 mg/kg | PP-281 |
| Thallium (Tl) | 15,0 mg/kg | PP-281 |
| Vanadium (V) | 0,3 mg/kg | PP-281 |
| Aluminum (Al) | 3,0 mg/kg | PP-281 |
| Arsenic (As) | 0,500 mg/kg | PP-281 |
| Nickel (Ni) | 0,6 mg/kg | PP-281 |
| Chromium (Cr) | 0,3 mg/kg | PP-281 |
| Cadmium (Cd) | 0,2 mg/kg | PP-281 |
| Copper (Cu) | 0,4 mg/kg | PP-281 |
| Iron (Fe) | 4,00 mg/kg | PP-281 |
| Manganese (Mn) | 0,2 mg/kg | PP-281 |
| Zinc (Zn) | 3,00 mg/kg | PP-281 |
| Cobalt (Co) | 0,7 mg/kg | PP-281 |
| Lead (Pb) | 1,5 mg/kg | PP-281 |
| **Biomolecules and physicochemical properties** | | |
| Total carbohydrates | 0,20 % | PE-358 |
| Total proteins | 0,2 % | PE-987 |
| Total lipids | 0,20 % | PE-356 |

**Table SI.1A. Summary of fertilization treatments applied during the 14-weeks greenhouse experiment.**

| Treatment | Application Mode | Dose per Application | Dilution | Frequency | Total Duration | Replication |
|---|---|---|---|---|---|---|
| SBLB | Alternating foliar and soil | Foliar: spray to runoff, 150 mL; Soil: 300 mL per plant | Foliar: 5% (v/v); Soil: undiluted | Every 15 days (alternating foliar and soil application, one week each) | 14 weeks | 5 trays × 4 plants (n = 20) |
| Chemical fertilizer (N: P: K 20:20:20) | Soil application only | 57 grams per plant | - | Every 15 days | 14 weeks | 5 trays × 4 plants (n = 20) |
| Control | Irrigation only | — | — | Same irrigation schedule | 14 weeks | 5 trays × 4 plants (n = 20) |

**Table SI.2:** Elemental composition of raw Sargassum biomass and solid residue obtained after anaerobic fermentation.

| Parameters | Result | | Unit |
|---|---|---|---|
| | *Sargassum* biomass | *Sargassum* fermentation wastes | |
| **Alkali and alkaline earth metals** | | | |
| Barium (Ba) | 30 | 89 | mg·kg⁻¹ |
| Beryllium (Be) | <0.05 | < 0.05 | mg·kg⁻¹ |
| Potassium (K) | 36,267 | 11,837 | mg·kg⁻¹ |
| Calcium (Ca) | >50,000 | 42,889 | mg·kg⁻¹ |
| Magnesium (Mg) | 10,071 | 3,612 | mg·kg⁻¹ |
| Sodium (Na) | 11,192 | 1,155 | mg·kg⁻¹ |
| **Heavy metals and metalloids** | | | |
| Mercury (Hg) | <0.5 | < 0.5 | mg·kg⁻¹ |
| Thallium (Tl) | <15.0 | < 15.0 | mg·kg⁻¹ |
| Vanadium (V) | 5 | 9.5 | mg·kg⁻¹ |
| Aluminum (Al) | 33 | 165 | mg·kg⁻¹ |
| Arsenic (As) | 72.4 | 8 | mg·kg⁻¹ |
| Nickel (Ni) | 3 | < 0.6 | mg·kg⁻¹ |
| Chromium (Cr) | 1 | 2.5 | mg·kg⁻¹ |
| Cadmium (Cd) | 1 | 0.4 | mg·kg⁻¹ |
| Copper (Cu) | 8 | 37 | mg·kg⁻¹ |
| Iron (Fe) | 82.2 | 1,007 | mg·kg⁻¹ |
| Manganese (Mn) | 24 | 30.3 | mg·kg⁻¹ |
| Zinc (Zn) | 7.44 | 43 | mg·kg⁻¹ |
| Cobalt (Co) | <0.7 | 2 | mg·kg⁻¹ |
| Molybdenum (Mo) | <1.2 | 1.4 | mg·kg⁻¹ |
| Lead (Pb) | <1.5 | < 1.5 | mg·kg⁻¹ |

**Table SI.3:** The limit of quantification in soils.

| Element | The limit of Quantifications | Method |
|---|---|---|
| **Macronutrients and micronutrients** | | |
| Phosphorus (P) | 0,60 mg/kg | EPA Method 3050B Rev.2 (1996) / EPA Method 6020B Rev.2 (2014) |
| Nitrogen (N) | 155 mg/kg | PEC-034 |
| Potassium (K) | 10,0 mg/kg | EPA Method 3050B Rev.2 (1996) / EPA Method 6020B Rev.2 (2014) |
| Calcium (Ca) | 10,00 mg/kg | EPA Method 3050B Rev.2 (1996) / EPA Method 6020B Rev.2 (2014) |
| Magnesium (Mg) | 0,3 mg/kg | EPA Method 3050B Rev.2 (1996) / EPA Method 6020B Rev.2 (2014) |
| Sodium (Na) | 1,00 mg/kg | EPA Method 3050B Rev.2 (1996) / EPA Method 6020B Rev.2 (2014) |
| Iron (Fe) | 0,010 mg/kg | EPA Method 3050B Rev.2 (1996) / EPA Method 6020B Rev.2 (2014) |
| Manganese (Mn) | 1,00 mg/kg | EPA Method 3050B Rev.2 (1996) / EPA Method 6020B Rev.2 (2014) |
| Copper (Cu) | 0,030 mg/kg | EPA Method 3050B Rev.2 (1996) / EPA Method 6020B Rev.2 (2014) |
| Zinc (Zn) | 0,140 mg/kg | EPA Method 3050B Rev.2 (1996) / EPA Method 6020B Rev.2 (2014) |
| Cobalt (Co) | 0,0080 mg/kg | EPA Method 3050B Rev.2 (1996) / EPA Method 6020B Rev.2 (2014) |
| Molybdenum (Mo) | 0,002 mg/kg | EPA Method 3050B Rev.2 (1996) / EPA Method 6020B Rev.2 (2014) |
| **Heavy metals and metalloids** | | |
| Arsenic (As) | 0,010 mg/kg | EPA Method 3050B Rev.2 (1996) / EPA Method 6020B Rev.2 (2014) |
| Cadmium (Cd) | 0,0008 mg/kg | EPA Method 3050B Rev.2 (1996) / EPA Method 6020B Rev.2 (2014) |
| Chromium (Cr) | 0,0080 mg/kg | EPA Method 3050B Rev.2 (1996) / EPA Method 6020B Rev.2 (2014) |
| Nickel (Ni) | 0,020 mg/kg | EPA Method 3050B Rev.2 (1996) / EPA Method 6020B Rev.2 (2014) |
| Lead (Pb) | 0,0020 mg/kg | EPA Method 3050B Rev.2 (1996) / EPA Method 6020B Rev.2 (2014) |

**Table SI.4:** The limit of quantification in vegetative tissues.

| Element | Limit of quantification | Method |
|---|---|---|
| **Arsenic (As)** | 0,01 mg/kg | ICP-MS |
| **Cadmium (Cd)** | 0,010 mg/kg | ICP-MS |
| **Chromium (Cr)** | 0,03 mg/kg | ICP-MS |
| **Copper (Cu)** | 0,03 mg/kg | ICP-MS |
| **Iron (Fe)** | 0,2 mg/kg | ICP-MS |
| **Manganese (Mn)** | 0,025 mg/kg | ICP-MS |
| **Lead (Pb)** | 0,010 mg/kg | ICP-MS |
| **Zinc (Zn)** | 0,50 mg/kg | ICP-MS |
| **Phosphorus (P)*** | 0,020 % | ICP-OES |
| **Nitrogen (N)*** | 0,50 % | NIRS |
| **Potassium (K)*** | 0,02 % | ICP-OES |
| **Calcium (Ca)*** | 0,03 % | ICP-OES |
| **Magnesium (Mg)*** | 0,038 % | ICP-OES |
| **Sodium (Na)** | 250 mg/kg | ICP-MS |

**Note:** Values marked with an asterisk (*) are expressed as a percentage (%) on a dry weight basis. All other elements are expressed in $mg \cdot kg^{-1}$.

**Table SI.5:** The limit of quantification in tomato fruits.

| Element | The limit of quantification | Method |
|---|---|---|
| **Alkali and alkaline earth metals** | | |
| Barium (Ba) | 0,12 mg/kg | PP-281 |
| Beryllium (Be | 0,05 mg/kg | PP-281 |
| Potassium (K) | 85,0 mg/kg | PP-281 |
| Calcium (Ca) | 33,0 mg/kg | PP-281 |
| Magnesium (Mg) | 31,0 mg/kg | PP-281 |
| Sodium (Na) | 26,0 mg/kg | PP-281 |
| **Heavy metals and metalloids** | | |
| Mercury (Hg) | 0,500 mg/kg | PP-281 |
| Thallium (Tl) | 15,0 mg/kg | PP-281 |
| Vanadium (V) | 0,3 mg/kg | PP-281 |
| Aluminum (Al) | 3,0 mg/kg | PP-281 |
| Arsenic (As) | 0,500 mg/kg | PP-281 |
| Nickel (Ni) | 0,6 mg/kg | PP-281 |
| Chromium (Cr) | 0,3 mg/kg | PP-281 |
| Cadmium (Cd) | 0,2 mg/kg | PP-281 |
| Copper (Cu) | 0,4 mg/kg | PP-281 |
| Iron (Fe) | 4,00 mg/kg | PP-281 |
| Manganese (Mn) | 0,2 mg/kg | PP-281 |
| Zinc (Zn) | 3,00 mg/kg | PP-281 |
| Cobalt (Co) | 0,7 mg/kg | PP-281 |
| Lead (Pb) | 1,5 mg/kg | PP-281 |
| **Biomolecules and physicochemical properties** | | |
| Total carbohydrates | 0,20 % | PE-358 |
| Total proteins | 0,2 % | PE-987 |
| Total lipids | 0,20 % | PE-356 |

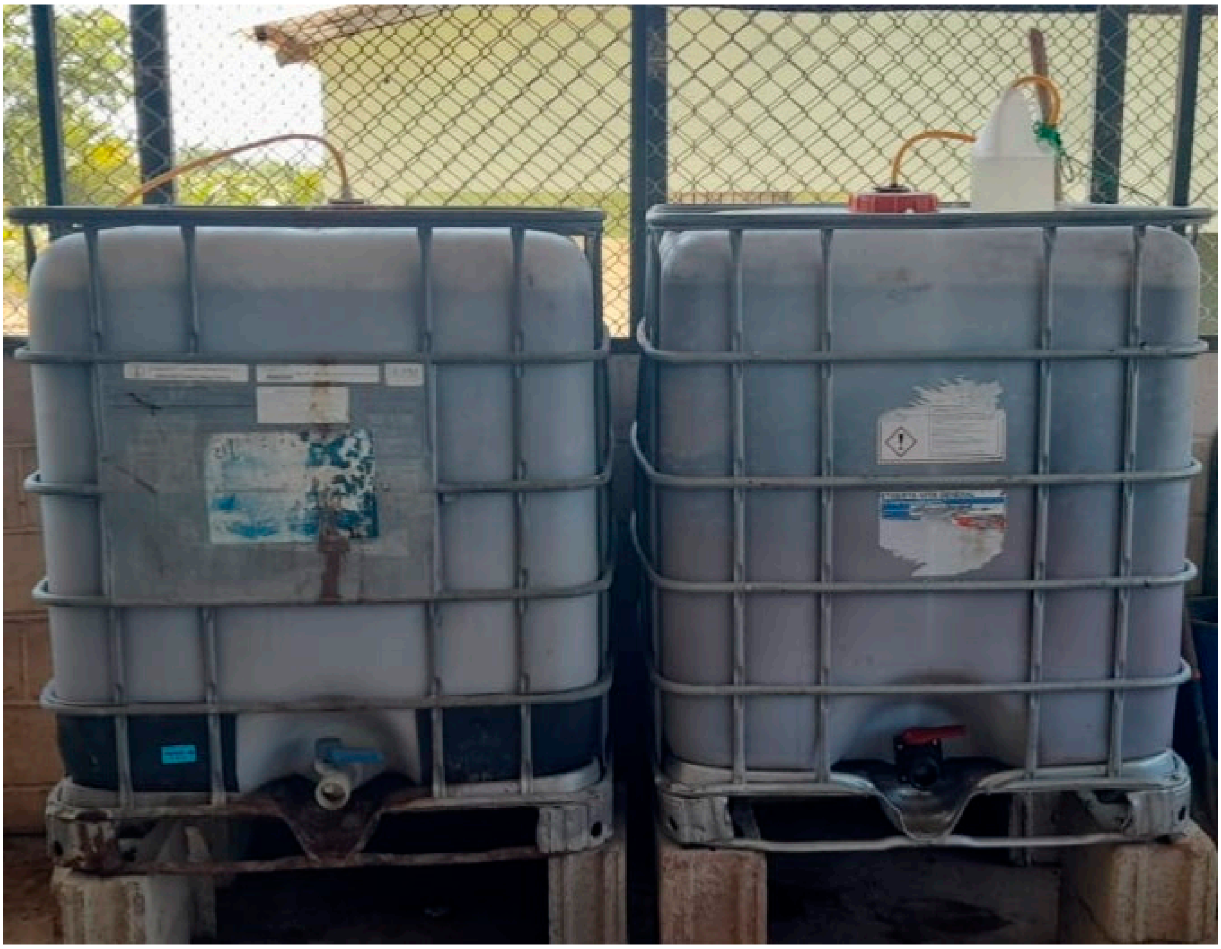

**Figure SI.1** One-cubic-meter cube fermeter at Banelino Bio-ferments Plant, in Hato del Medio Arriba, Monte Cristi, Dominican Republic.
